# Introducing Minimally Invasive Tissue Sampling to Ascertain Cause of Death in Children and Stillbirths in Central Mozambique

**DOI:** 10.4269/ajtmh.25-0253

**Published:** 2026-02-03

**Authors:** Inácio Mandomando, Anélsio Cossa, Augusto Messa, António Sitoe, Pio Vitorino, Amílcar Magaço, Rita Mabunda, Alexandre Macanze, Judice Miguel, Izete Figueiredo, Agbessi Amouzou, Robert Black, Almamy Malick Kante, Elísio Xerinda, Sergio Massora, Marcelinio Garrine, Percina Chirinda, Placido Assane, Ivalda Macicame, Dercio Jordao, Ladino Suade, Celisa Mendonça de Assis, Arsénia Massinga, Maria Maixenchs, Ariel Nhacolo, Khatia Munguambe, Portia Mutevedzi, Robert F. Breiman, Cynthia G. Whitney, Diana M. Blau, Mischka Garel, Jaume Ordi, Natalia Rakislova, Quique Bassat

**Affiliations:** ^1^ Centro de Investigação em Saúde de Manhiça, Maputo, Mozambique;; ^2^ ISGlobal, Barcelona, Spain;; ^3^ Instituto Nacional de Saúde, Maputo, Mozambique;; ^4^ Global Health and Tropical Medicine, Louisiana-REAL, Instituto de Higiene e Medicina Tropical, Universidade NOVA de Lisboa, Lisboa, Portugal;; ^5^ Facultat de Medicina i Ciències de la Salut, Universitat de Barcelona, Barcelona, Spain;; ^6^ Johns Hopkins University, Baltimore, Maryland;; ^7^ Hospital Central de Quelimane, Quelimane, Mozambique;; ^8^ Emory Global Health Institute, Emory University, Atlanta, Georgia;; ^9^ Centers for Disease Control and Prevention, Atlanta, Georgia;; ^10^ ICREA, Barcelona, Spain;; ^11^ Institut Clínic de Medicina I Dermatologia, Hospital Clínic de Barcelona, Barcelona, Spain;; ^12^ Pediatrics Department, Hospital Sant Joan de Déu, Universitat de Barcelona, Barcelona, Spain;; ^13^ CIBER de Epidemiología y Salud Pública, Instituto de Salud Carlos III, Madrid, Spain

## Abstract

Minimally invasive tissue sampling (MITS) has been used as an alternative to complete autopsy to track causes of death (CoDs) in South Asia and sub-Saharan Africa as part of the Child Health and Mortality Prevention Surveillance program. However, community acceptance, rapid identification of deaths, and adequate functional laboratory infrastructures (e.g., pathology, conventional microbiology, and molecular microbiology) are critical for successful implementation. We describe the experience of implementing MITS in an urban district with socioeconomic and cultural diversity in Zambézia Province, central Mozambique. For successful implementation of mortality surveillance using MITS, high-level advocacy involving the Provincial Government and all stakeholders as well as engagement and sensitization of all segments of the communities, including traditional healers, community leaders, and mass media, were critical for the acceptability of the procedure. Additionally, social and behavior studies were conducted to assess perceptions, sociocultural factors, acceptability, and feasibility of the MITS procedure. These studies helped adapt the MITS protocol to the local context to minimize the risk of misunderstanding the mortality surveillance using MITS procedures. There was significant investment in capacity building, including financial support for laboratory equipment acquisition and maintenance, reagents, and consumables required for microbiological screening protocols of MITS and to support the needs for diagnostics of patients with severe disease seeking care. Experiences from Quelimane and other sites and data generated in the Countrywide Mortality Surveillance for Action to support evidence-based decision-making processes on health policy were critical for the community to understand the benefit of determining young children CoD to guide future interventions.

## INTRODUCTION

The Child Health and Mortality Prevention Surveillance (CHAMPS) program was established in 2015 as a multisite mortality surveillance network aiming to track causes of death (CoDs) in stillbirths and children younger than 5 years old in high-mortality areas (>50 deaths per 1,000 live births) in sub-Saharan Africa and South Asia.^1^ As its core methodological innovation, CHAMPS uses the novel minimally invasive tissue sampling (MITS) approach to obtain postmortem samples of key tissues and body fluids that are amenable to comprehensive laboratory and pathology investigations.^2^ Child Health and Mortality Prevention Surveillance sites (hospital and community-based surveillances areas) have been established across the CHAMPS network to document all deaths in children younger than 5 years old and stillbirths occurring in the catchment areas, usually a whole district. Families are approached within the first 24–36 hours after their child’s death, and they are asked to give consent for MITS and the standardized investigative procedures.^3^

Currently, the network includes well-established sites in seven African countries (Ethiopia, Kenya, Mali, Mozambique, Nigeria, Sierra Leone, and South Africa) and two in South Asia (Bangladesh and Pakistan).^4^ To conduct MITS, the sites must have adequate functional laboratory infrastructures (i.e., pathology, conventional microbiology, and molecular microbiology) as postmortem studies rely on accurate laboratory diagnosis.^5^^,^^6^

In Mozambique, CHAMPS has been implemented since 2016 by the Centro de Investigação em Saúde de Manhiça (CISM), which is located in a rural district of southern Mozambique, in a well-defined catchment area with approximately 210,000 inhabitants under rigorous and continuous demographic surveillance.^7^^,^^8^ Since 2018, with support from the Bill & Melinda Gates Foundation, the Instituto Nacional de Saúde (INS [National Health Institute]) in partnership with the Instituto Nacional de Estatística (INE [National Institute for Statistics]) and the Johns Hopkins University (Baltimore, MD) have implemented Countrywide Mortality Surveillance for Action (COMSA) in Mozambique. Countrywide Mortality Surveillance for Action is a nationwide sample registration surveillance system capturing community vital events (pregnancies, pregnancy outcomes, and deaths) and CoD data using verbal autopsies. The system aims to generate reliable annual statistics on mortality and CoD for all ages across the country.^9^

Verbal autopsies rely on interviews with a relative of the deceased, but they have a low accuracy in assigning the CoD at the individual level as described elsewhere.^10^ Therefore, MITS-based CoD data using advanced histopathology and microbiology procedures are used to calibrate the verbal autopsy data generated by the COMSA project.^11^ To increase the number of MITS cases produced in Mozambique to support the calibration, CHAMPS collaborated with the COMSA project to extend the MITS procedures to the Hospital Central de Quelimane (HCQ) in the district of Quelimane, which is the urban capital of Zambézia Province. Thus, CISM and the Barcelona Institute for Global Health were responsible for the implementation of COMSA and later, a new CHAMPS site in central Zambézia, Mozambique.

## SITE SELECTION FOR MITS IMPLEMENTATION IN THE CONTEXT OF COMSA

The site selection process for COMSA was aligned with the site selection criteria used by CHAMPS, which included 1) mortality rate >50/1,000 live births for children younger than 5 years old, 2) adequate infrastructures for mortality surveillance, and 3) sufficient laboratory capacities (pathology and microbiology). Initially, two provinces (Nampula and Sofala) were short-listed as potential sites to implement MITS as part of the COMSA project. However, the existence of a newly inaugurated referral hospital in 2016 (HCQ) equipped with basic pathology and bacteriology laboratories together with the fact that Zambézia Province had a high prevalence of malaria (34.9%) and HIV (17.1%) among the adult population with high associated child mortality (*n* = 109/1,000 live births in 2017) ranked Quelimane as the most appropriate catchment area for conducting MITS.^9^^,^^12^^,^^13^

Postmortem studies are culturally sensitive as the communities understand that the procedure requires opening the body of the deceased and extracting key organs or fragments, which may conflict with local beliefs and practices. In this context, high-level engagement and advocacy at different levels, including the local governors, stakeholders, and the community (community leaders, community representatives [including women of reproductive age], political parties, civil society, and media), were identified as a crucial first step for the successful acceptance of the MITS procedure.^3^ This was particularly relevant in an area like the province of Zambézia and especially in Quelimane city, which is characterized by a marked sociocultural diversity, where misunderstandings and misinformation on health-related programs have been previously reported.^14^^,^^15^

## PARTNERSHIP AND ENGAGEMENT OF THE LOCAL COMMUNITY

Before advocating for mortality surveillance using MITS, a partnership between the CISM, the INS, and the INE was established, and the investigators of the three institutions visited the province of Zambézia, specifically its capital city Quelimane, to conduct a site assessment. Zambézia visits included meetings with provincial and community leaders and hospital (central and general) visits to select the most appropriate hospital to implement MITS. Meetings were held with the Zambézia Provincial Directorate of Health, the Provincial Governor, and other key stakeholders: Directors of the Quelimane Central and General Hospitals, community and religious leaders (including traditional healers), and political parties represented in the provincial assembly (Frente de Libertação de Moçambique, Resistência Nacional de Moçambique, and Movimento Democrático de Moçambique). These meetings were conducted between October 2017 and March 2018, involving the CHAMPS team (Mozambican site and Emory Global Health Institute investigators) as well as investigators from the INE and the INS. The main goals of these meetings were to inform and share information about the rationale, objectives, and study design; provide an overview of CHAMPS as a useful platform to ascertain the CoD; and explain how MITS is performed, with specific emphasis of its minimally invasive nature (in comparison with the complete diagnostic autopsy) and tools required (needles and syringes).^16^

Furthermore, specific meetings with representatives of the general media operating in the province were held to explain the key activities to be implemented in COMSA and the hospital-based mortality surveillance. During these meetings, the MITS procedure, the place for laboratory testing (Quelimane, Manhiça, or the CDC in Atlanta, GA) and the process of giving feedback on the CoD to the families were explained and discussed.

The ultimate goal of this partnership and engagement was ensuring that the concept of MITS and its procedure was clear for the stakeholders, media, politicians, and community leaders who are in regular contact with the community to avoid misunderstandings and miscommunication about the postmortem surveillance that per se is sensitive because it requires the manipulation of bodies of deceased children to collect small tissue fragments and body fluids for laboratory analysis. Lastly, the study team tried to anticipate potential feasibility and acceptability for conducting postmortem investigations of CoDs.

The study team received feedback from all groups on the best way to approach families of deceased children for consent and to offer opportunities for the families to watch the MITS procedure. The inputs and feedback provided were also relevant to design an appropriate strategy for assessing the community acceptability and feasibility of conducting MITS in the Quelimane district. This city is characterized by high cultural diversity, where a history of myths (e.g., the bloodsucker phenomenon, which is based on the local belief that “strange” individuals, usually unknown people or foreigners to the specific community, enter huts at night to suck people’s blood using special tools),^17^ rumors, negative perceptions, and potential reputational issues of specific public health initiatives and interventions were known to exist and had resulted in, for example, lower uptake of HIV service and treatment.^17^

### Social behavior science.

#### Formative research.

In preparation for the COMSA MITS study, community sensitization activities were initiated in August 2018; a series of interactive workshops with community leaders and members was held using an adaptation of the approach known as participatory rural appraisal called Participatory Inquiry into Community Knowledge on Child Health and Mortality Prevention (PICK-CHAMPS).^18^ The workshop sessions included community leaders, religious leaders, nharrubes (traditional authorities tasked with the washing of the bodies before the funeral), matronas (traditional birth attendants), community members, and other local influential people. The goal of the workshops was to get insights from the community about the feasibility of performing MITS in the cultural context of Quelimane and to get initial perspectives on alignments and tensions between MITS procedures and community perceptions and priorities.

The participants’ feedback from PICK-CHAMPS revealed moderate levels of alignment between MITS activities and community priorities, which were prevalent in the same proportions as tension with local beliefs, especially for communities in which rumors and myths about “bloodsuckers” are rife.^19^ The formative research concluded that MITS was considered a positive innovation to determine the CoDs, although community members remain skeptical about the procedure because of tensions with religion and tradition. Therefore, the implementation of MITS in Quelimane should prioritize the involvement of a variety of influential community and religious leaders.^20^ These findings were used for designing the strategy for community engagement.

#### Community engagement.

After the conclusion of the PICK-CHAMPS activities, we proceeded with a series of community engagement initiatives. These efforts involved comprehensive meetings with diverse community stakeholders, and they emphasized the engagement of individuals who did not partake in the PICK-CHAMPS sessions. This inclusive approach included the previously identified groups of community leaders, religious leaders, nharrubes, matronas, and community members so as to help disseminate the information in their communities. These sessions served as a platform to introduce and familiarize participants with the goals and methodologies of CHAMPS and MITS. Moreover, these meetings provided an invaluable opportunity to delve into local norms and customs that hold significance within the context of implementing MITS effectively.

The overarching aims were not solely to present COMSA and introduce the MITS procedure but also, to create strong partnerships and alliances with the community (community leaders, key informants, nharrubes, matronas, and women of reproductive age). These partnerships have been strengthened as CHAMPS endeavors to devise and implement sustainable, low-cost initiatives to address the communities’ needs related to maternal and child health. Establishing these partnerships was crucial to ensuring that implementation of MITS aligns seamlessly with the community’s values and practices. Through this partnership, we sought to cultivate a collaborative environment wherein the community becomes an integral and active participant in the process, fostering understanding and cooperation between all participants.

#### Actionable recommendation for MITS.

Based on the formative research, the actionable recommendations for the seamless integration of MITS predominantly revolved around honoring and integrating local cultural norms, especially regarding respect for the deceased children. Foremost among these recommendations was the need for the MITS approach to be adapted to prevent any undue delay in the burial process. This approach not only honored the cultural practices surrounding the deceased, but also, it demonstrated a sensitive and respectful engagement with the community’s traditions. Minimally invasive tissue sampling was not performed for families approached that reported a hurry to bury the bodies.

Equally fundamental was the recommendation to actively involve family members and community leaders in the MITS procedure. This inclusive approach bridged the gap between medical protocols and cultural sensitivities. By involving and seeking consent before carrying out MITS (which are mandatory for ethical reasons), inviting families and community leaders to observe the MITS procedure, and providing a thorough explanation of how the MITS is carried out, the COMSA team established trust, transparency, and respect for the wishes of the bereaved.

## BUILDING CAPACITY FOR MORTALITY SURVEILLANCE USING THE MITS APPROACH

### Hospital mortality surveillance.

No comprehensive mortality surveillance system existed before COMSA MITS was established at the HCQ. However, the Ministry of Health requested “mortality audits” of deaths in all hospitals, even though nearly all lacked diagnostic testing data. The lack of a functioning laboratory to determine the contribution of specific pathogens in the definitive CoD was one of the limitations found in many hospitals, including the HCQ before the introduction of MITS.

The MITS approach requires timely reporting of a death to allow for sample collection within 24–36 hours (36 hours if the body is refrigerated) and before burial.^3^ To enhance mortality surveillance at the central hospital, we mapped all of the facilities and/or wards where a child death could occur. We established a timely reporting system for notification of all deaths through a call center, allowing the study team to assess the eligibility criteria for MITS as previously described so that the COMSA team could approach the families of the deceased child for timely consent and performance of MITS.^20^ This included the development and implementation of standard procedures for notification of deaths in the first 24–36 hours, obtaining consent of families, data collection, and MITS for eligible cases.

Reporting of deaths is done through a 24-hours-a-day, 365-days-a-year toll-free number (“green line”). The call center officer is in charge of evaluating whether a reported death meets inclusion and exclusion criteria. We held numerous meetings to engage health care professionals from the Department of Pediatrics who were responsible for reporting all deaths occurring in the department to the COMSA team on time. To reinforce this, we created some leaflets with the call center numbers and posted them in all notification sectors of the Department of Pediatrics. To ensure that deaths did not go unreported, we also established twice-daily supervision visits to each notifying sector of the Department of Pediatrics. Every 15 days, we carried out surveys of deaths in the notification sectors to assess how well the records matched notifications.

#### Facility to perform MITS.

To guarantee the minimal needs for carrying out MITS in a suitable environment, we conducted a needs assessment; we improved the MITS room by replacing the floor with a more suitable one (white porcelain tiles) and acquiring specific equipment, such as an air conditioner, a mobile autopsy table, analytical and electronic scales, a wooden meter for height measurement, a refrigerator for preserving placentas, a printer, and a camera. In addition, to minimize crosscontamination of samples, we improved the legal medical autopsy room so that the MITS room would no longer be used for both MITS and full forensic autopsies. The sample collection team was trained in good clinical and laboratory practice standards as well as the MITS technique. Hospital employees (morgue attendants, nurses, doctors, and assistants from reporting sectors) were also trained on good clinical practices, biosafety, and MITS procedures. The sample collection team was trained in registering the data on the Research Electronic Data Capture (REDCap) platform (the disposition of body form and the MITS specimen collection form), taking photographs, associating the photographs with each case file, and scanning and storing clinical records for subsequent clinical data abstraction.^21^

#### Laboratory capacities.

The availability of laboratory infrastructure and capacity was one of the key factors for the decision to select Quelimane to implement MITS in the context of COMSA. The HCQ had a laboratory composed of different departments, including microbiology, pathology, parasitology, biochemistry, and hematology. However, similar to many other laboratories in low- and middle-income countries, there was a lack of quality assurance systems, which are critical for ensuring laboratory data quality based on international standards; a quality assurance system was implemented to respond to the needs of MITS (in 2019), and it is also used for the general microbiology laboratory. Other gaps at the time of site assessment included 1) the lack of records of regular maintenance of the equipment; 2) the lack of laboratory consumables and reagents; and 3) regular power failures because of fluctuations in the national electricity network, resulting in recurring damage of equipment. Details of the situational analysis of the capacities available and improvements made are described by area.

#### Bacteriology.

The primary objective of the bacteriology laboratory of the HCQ is to support clinicians with accurate bacteriological diagnosis (culture, isolation, identification, and antimicrobial susceptibility testing of bacterial pathogens) from clinical specimens using conventional microbiology techniques. The semiautomated BACT/ALERT^®^ 3D 60 (Biomerieux, Marcy I’Etoile, France) machine was available in the bacteriology laboratory, but it was not operational because the technicians were not trained on how to use it; therefore, blood cultures were incubated manually. There were no standard operating procedures (SOPs) in place; in addition, there were inadequate quality control practices, human blood was used for preparing blood-containing media, and there was a lack of internal and external quality control, including American Type Culture Collection (ATCC) strains. The use of human blood is contraindicated because it may contain antibodies and antimicrobial agents, which may also inhibit growth or cause false hemolysis apart from the risk of exposure to HIV and other blood-borne infections.^22^^,^^23^

To improve the laboratory quality, SOPs, manuals, and guidelines were developed for different procedures, such as for sample collection and processing, biosafety, and other topics. In addition, a quality assurance program was also established, including the setup of internal quality control for media preparation, reagents check, and antimicrobial susceptibility testing using ATCC strains. These changes improved the reliability of microbiology results, making the blood culture procedure available to patients admitted with severe disease and suspected of sepsis.

Like many other sub-Saharan African countries, staff shortage and high turnover are critical issues, particularly in public laboratories. Before COMSA, all bacteriological activities were supported by only two laboratory technicians, and with COMSA resources, two additional technicians were hired to support both routine and MITS-related sample processing, contributing to the expansion of the time of laboratory operation from 5 to 8 hours daily.

To improve competency for the laboratory technicians, we provided on-site or external training (in Manhiça) for the staff, which included good clinical laboratory practice, biosafety, laboratory protocols for Gram staining, bacterial culture, bacterial identification, and antimicrobial susceptibility testing, under the supervision of microbiologists from Manhiça. Training on malaria smear reading for parasite quantification was also conducted, and competent technicians were certified, which reduced the turnaround time for results delivery because initially, malaria smears were shipped to Manhica for reading and parasite quantification. Technicians were certified as competent after comparing their results with a referee reader.

Furthermore, an exchange training program (1–4 months) was instituted between Manhiça and Quelimane as part of local capacity and expertise building to respond to the demands of COMSA, and future studies were planned at that time, benefiting the local staff. A competency-based training program held at CISM laboratory units and clinical facilities through a rotation model provides exceptional services through professional collaboration among laboratory technologists, technical specialists, assistants, and the MITS team. During the rotation, trainings were held on MITS sample processing, handling of biohazardous material, laboratory records and results management, ordering and control of stocks of laboratory supplies, sample storage, and shipment (according to International Air Transport Association [IATA] regulations). Discussions around the laboratory services milestones were held to provide tools to easily identify potential constraints and solutions, reduce the turnaround time of results, and enhance the overall performance of the HCQ Laboratory. In addition, there was a plan designed for the coexistence of the two CHAMPS sites until an eventual transition phase; eventually, the Manhiça site would be phased out, and only the Quelimane site would be maintained.

#### Pathology.

The histopathology laboratory of the HCQ was one of the best-equipped laboratories in the area for conducting routine activities to support the clinical diagnosis of patients seeking care. However, some equipment (e.g., a teaching microscope and a chemical fume hood) relevant or crucial for the study was unavailable. Despite being well equipped, no routine activities were undertaken at the time of site assessment because of 1) lack of laboratory consumables and reagents, 2) equipment that had not been tested or serviced since installation, 3) staff who were not trained on how to use some equipment, and 4) the presence of only one laboratory technician from the hospital to support the pathologist.

To overcome these gaps, we improved the laboratory capacity using COMSA resources by purchasing additional equipment, laboratory consumables, and reagents; servicing key equipment; and providing training to the technicians on how to operate them. Laboratory consumables and reagents were acquired to support not only the needs of the COMSA study but also the processing of routine samples. Furthermore, staffing was reinforced by COMSA hiring a laboratory technologist to supervise the day-to-day pathology activities of CHAMPS as well as the processing of routine samples from the hospital for clinical management.

Two technicians (one from the hospital and the second one hired specifically for COMSA) were trained in Barcelona, Spain to implement pathology activities at the HCQ. The technicians were also trained in the preparation of histological sections, ensuring the precision and integrity of the samples during the process. In addition, technicians were also trained on how to prepare the tissues samples on paraffin blocks, and they learned how to properly embed samples in paraffin to facilitate sections and preserve cellular structure. Performing stains, such as hematoxylin and eosin, was another key part of the training, allowing technicians to visualize different cellular and tissue components clearly. Furthermore, technicians were introduced to some special techniques, such as Giemsa or Ziehl–Nielsen stains, which can be useful in specific cases of pathological analysis.

### Infrastructures and equipment.

With regard to infrastructure, the recommendations from the site assessment report noted a need for substantial investment to accommodate the minimal requirements for the study. These requirements included the acquisition of laboratory equipment for both the bacteriology and pathology laboratories (e.g., −80°C and −20°C freezers, refrigerators, CO_2_ incubators, level II biosafety cabinets, and an autopsy table to conduct MITS among others). Additionally, electric power system upgrades, including a backup generator and an uninterruptible power supply system to ensure the continuous supply of electricity in case of power failure, were purchased and installed at the hospital to serve the laboratories and other critical areas of the hospital (e.g., nursery and surgery) because of the unreliability of the power supply.

**Figure 1.**
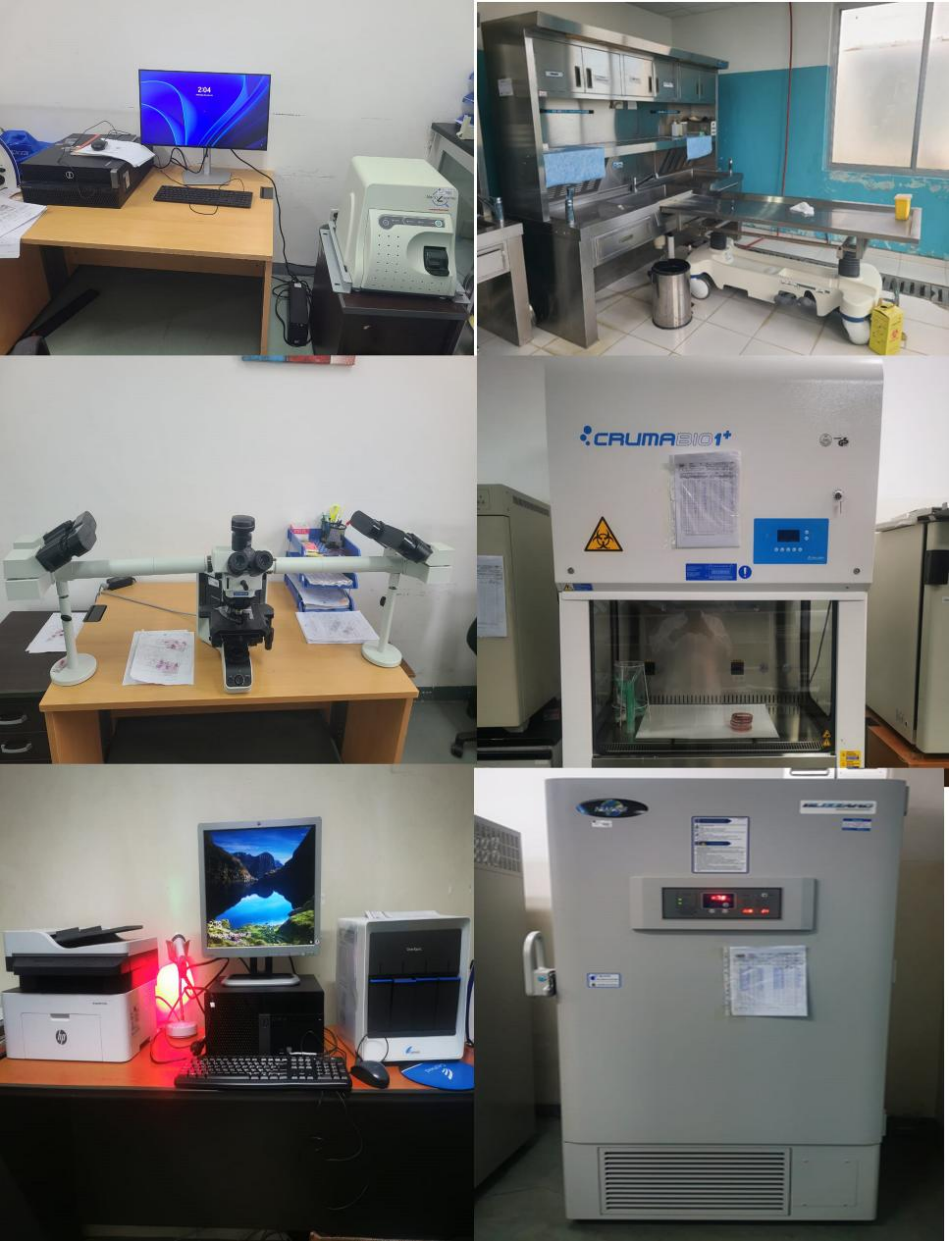
Pictures of improvements of laboratory capacities at Hospital Central de Quelimane in central Mozambique.

Furthermore, repair and maintenance services were procured for all of the equipment used for CHAMPS and serving other patients in critical areas. These services also included the improvement of internet connectivity for communication and real-time upload of the data, including telepathology sessions. Figure 1 shows pictures of the equipment purchased to improve the laboratory capacity at the HCQ.

## CHALLENGES FOR IMPLEMENTING MITS IN QUELIMANE

Multiple challenges to setting up this work were identified, including buy-in to the new concept of MITS in the community with little exposure to research activities, the lack of laboratory equipment and maintenance contracts, the lack of consumables and reagents, and the absence of quality assurance to ensure that laboratory results were trustable. All of these challenges required specific funds, which were provided by the Bill & Melinda Gates Foundation through COMSA and CHAMPS grants. Last and not least, difficulties in following up with the families (because the references that the families provided during the enrollment were not consistent with the locations, few families had cell phone contacts, and there was no demographic surveillance system) for the verbal autopsy interviews were among the main challenges and lessons learned to implement COMSA MITS in Quelimane. To overcome these challenges, we had to implement the collection of GPS coordinates in addition to an establishment of a demographic surveillance platform to support MITS and pregnancy surveillance.

## SUMMARY

For the successful implementation of mortality surveillance using the MITS approach in the Quelimane district, high-level advocacy involving the Provincial Government and all stakeholders followed by engagement and sensitization of all segments of the communities, including traditional healers and mass media, were critical to the acceptability of the procedure by the study community. More than 85% of families approached accepted the MITS procedure. Actionable recommendations from social behavior science studies assessing perception, feasibility, and acceptability of the MITS procedure were pivotal for the adaptation of the study protocol to the local context. The experiences learned from Manhiça and other sites, particularly the data generated in CHAMPS to support an evidence-based decision-making process on health policy, were critical for the community to understand the benefit of determining the CoD of the deceased children.
